# Thiopurine methyltransferase genotyping in Palestinian childhood acute lymphoblastic leukemia patients

**DOI:** 10.1186/2052-1839-13-3

**Published:** 2013-04-10

**Authors:** Basim Mohammad Ayesh, Wael Mohammad Harb, Abdalla Assaf Abed

**Affiliations:** 1 Medical Technology Department, Al Aqsa University, Gaza, Palestinian authority; 2 MOH, Shouhada AL-Aqsa Hospital Laboratory, Gaza, Palestinian authority; 3 Biology Department, Islamic University of Gaza, Gaza, Palestinian authority

**Keywords:** Thiopurine S-methyltransferase, TPMT, Acute lymphoblastic leukemia

## Abstract

**Background:**

The genetic polymorphism of thiopurine methyltransferase (TPMT) is well characterized in most populations. Four common polymorphic alleles are associated with impaired activity of the enzyme. These are TPMT*2 (238G>C), TPMT*3B (c.460G>A), TPMT*3A (c.460G>A and c.719A>G) and TPMT*3C (c.719A>G). The aim of the present study was to determine the frequency of TPMT polymorphisms and their association with the occurrence of adverse events, during 6-mercaptopurine therapy in pediatric acute lymphoblastic leukemic (ALL) patients in Gaza Strip.

**Methods:**

A total of 56 DNA samples from all pediatric ALL patients admitted to the pediatric hematology departments of Gaza strip hospitals were analyzed. Genomic DNA from peripheral blood leukocytes was isolated and the TPMT*2*,* TPMT*3B TPMT*3A and TPMT*3C allelic polymorphism was determined by PCR-RFLP and allele specific PCR technique.

**Results:**

No TPMT*2, *3B or *3C alleles were detected. Only one, out of 56 patients, was found heterozygous for the TPMT*3A allele. Thus, the frequency of TPMT*3A allele was calculated to be 0.89%. Fourteen patients of ALL were suffering from myelotoxicity during 6-MP therapy. From our results, no significant association could be established between clinical and laboratory data and/or the presence of the mutation in TPMT gene.

**Conclusion:**

TPMT*3A was the only deficiency allele detected in our population with an allelic frequency of 0.89%. Other polymorphic alleles in TPMT gene, or factors other than TPMT polymorphisms may be responsible for the development of myelosuppression in cases that don’t carry the investigated TPMT alleles (*2, *3A, *3B and *3C). Therefore, more studies are recommended to study such factors.

## Background

Thiopurine drugs have been widely used for the treatment of leukemia, autoimmune diseases and organ transplants. Oral 6-mercaptopurine (6-MP) is routinely used in maintenance treatment of acute lymphoblastic leukemia in children, which contributes to the high cure rates achieved [1,2]. Both 6-Mercaptopurine is a prodrug that requires activation by hypoxanthine-guanine phosphoribosyl transferase (HGPRT) to exert a cytotoxic effect [2]. Alternatively, this agent can undergo S-methylation catalyzed by thiopurine methyltransferase (TPMT) to 6-methylmercaptopurine (6-MeMP) or oxidation to thiouric acid via xanthine oxidase [3]. Metabolism via either TPMT or xanthine oxidase reduces formation of the active thioguanine nucleotides.

TPMT enzyme activity is largely influenced by polymorphisms in the TPMT gene. People heterozygous for TPMT mutations have intermediate activity while those homozygous for the mutation have low activity [4]. Over 23 variants of the TPMT gene are associated with decreased TPMT activity [5]. Three variant alleles, TPMT*2 (c.238G>C), TPMT*3A (c.460G>A and c.719A>G) and TPMT*3C (c.719A>G) account for 80–95% of intermediate or low activity cases [6].

Variation in TPMT activity regulates thiopurine toxicity and therapeutic efficacy of thiopurine drugs. Approximately 1 in 300 have low activity, 6-11% have intermediate activity and 89-94% have high activity [7-9].

TPMT is encoded by a 34 kb gene consisting of 10 exons and nine introns and has been localized to chromosome 6p22.3 [10].

According to the Palestinian Ministry of health hospital records, in Gaza Strip, the total number of recorded childhood ALL patients in 2006 was 15 patients, 14 patients in 2007, 15 patients in 2008 and 12 patients by August, 2009. 6-Mercaptopurine is administered for all patients at one stage or another of their treatment protocols.

No data are available about the state of TPMT allelic variation in Gaza neither testing of TPMT polymorphism is carried out. Therefore, this study was conducted.

## Methods

### Patients and sample collection

This is a descriptive study with Convenience sampling and definite time period. Fifty six children suffering from and being managed for acute lymphoblastic leukemia in European Gaza hospital, AL-Nasser hospital and Abd El Aziz Al Rantisi hospital were included in the period between July 2008 to august 2009. The study population included all patients attending these hospitals at time of the study. Other types of leukemia were excluded. Approximately 2.5 ml venous blood samples were collected, from the patients in EDTA tubes.

The procedures of the study were approved by the Helsinki research ethics committee of the Palestinian authority ministry of health according to the World Medical Association Declaration of Helsinki [11], and a written consent was obtained from the parents of each patient.

### TPMT genotyping

DNA was extracted from 300 μl whole blood samples using a commercial kit (promega, USA), according to the manufacture recommendations, and based on nonorganic extraction procedure. The extracted DNA was resuspended in 100 μl of the provided alkaline hydration solution at 65°C.

A total of 56 DNA samples were analyzed. Total genomic DNA extracted from peripheral leucocytes was processed by PCR either immediately or stored at -20°C until being used.

An allele-specific PCR [12] was used to analyze the mutation c.238G>C in exon 5 (TPMT *2 allele), using sequence specific primers (wild type specific: 5′-GTA TGA TTT TAT GCA GGT TTG-3′, mutant specific: 5′- GTA TGA TTT TAT GCA GGT TTC-3′ and common: 5′-TAA ATA GGA ACC ATC GGA CAC-3′) in two separate 20 μl reactions containing 0.5 μM of each primer, 5 μl DNA 1X PCR master mix (promega, USA). The cycling conditions consisted 35 cycles of 94°C for 30 s, 53°C for 30 s, and 72°C for 30 s.

The mutations c.719A>G in exon 10 and c.460G>A in exon 7 (TPMT*3A, TPMT*3B and TPMT*3C alleles) were determined by polymerase-chain reaction-restriction fragment length polymorphism analysis (PCR-RFLP) using *Acc*I and *Mwo*I respectively (New England Biolabs, USA) [12]. The PCR amplifications were carried out in 20 μl final volume containing 0.5 μM of the primers (5′-AAG TGT TGG GAT TAC AGG TG-3′ and 5′-TCC TCA AAA ACA TGT CAG TGT G-3′ for c.719A>G; and 5′-GGG ACG CTG CTC ATC TTC T-3′ and 5′-GCC TTA CAC CCA GGT CTC TG-3′ for c.460G>A), 5 μl DNA and 1X of PCR master mix (promega, USA). The cycling conditions consisted of 35 cycles of 94°C for 1 min, 56°C for 1 min, 72°C for 1 min for c.719A>G; and 35 cycles of 94°C for 1min, 59°C for 1 min, 72°C for 1 min for c.460G>A.

### Data collection and analysis

Relevant medical information was collected from the patient’s records in the hospitals. Data included adverse effects such as leucopoenia (white blood cells count <3000/ cubic millimeter), thrombocytopenia (platelets < 100000/ cubic millimeter), abnormal liver function (elevation of ALT level of 2 or more times the upper limit of normal); and duration of 6-MP therapy at time of sampling and at the time of adverse effects appearance. Data were analyzed using the SPSS software (Version. 17) as needed. Chi square test and One-Way ANOVA were used and P-values of < 0.05 were considered statistically significant. Means were presented ± standard deviation.

## Results

### Description of the study population

In this study, the sample included 56 pediatric patients suffering from ALL; 32 (57.1%) were males and 24 (42.9%) were females. Their ages at the time of diagnosis varied between 6 months to 12 years (mean 4.4 ± 2.6 years). The majority of patients were between 3.7 - 5.1 years with 95% Confidence interval. All patients were diagnosed as ALL patients by blood film, bone marrow aspiration and complete blood cell count (CBC).

In the present study the calculated incidence of ALL among children in Gaza strip during the specified period of samples collection was 2 patients per 100000 children. The incidence of ALL is higher in males than in females.

### Management of patients

During the collection of the sample, most of the patients (75%) were given 6-MP for different periods of time and 25% of them finished having the 6-MP treatment.

Among the 42 patients who were receiving 6-MP at the time of the sample collection, 6 patients suffered from leucopoenia (white blood cells count <3000/cubic millimeter); 5 patients from leucopoenia and thrombocytopenia; 3 patients from leucopoenia , thrombocytopenia and liver toxicity and 3 patients had only liver toxicity. When such complications took place, the only intervention was 6-MP dose reduction. During the course of management, one patient had neuroblastoma and one patient had mild brain atrophy, which likely didn’t result from the 6-MP treatment. Bone marrow transplantation was performed for 2 patients besides the 6-MP treatment.

### TPMT genotypes

The TPMT*2 allele (c.238G>C). TPMT*3B allele (c.460G>A) and TPMT*C allele (c.719A>G) were not detected in the studied patients, while one patient was heterozygous for the TPMT*3A allele (both c.460G>A and c.719A>G) with allelic frequency of 0.89% (Table 1).

**Table 1:** Frequencies of TPMT alleles in 56 samples of Gaza pediatric patient with ALL

**Allele**	**SNP**	**Amino acid substitution**	**Allelic count**	**Allelic frequency %**
TPMT*1	Wild-Type	**-**	**111**	**99.11**
TPMT*2	c.238G>C	Ala>Pro	**0**	**0**
TPMT*3A	c.460G>A, c.719A>G	Ala>Thr, Tyr> Cys	**1**	**0.89**
TPMT*3B	c.460G>A	Ala>Thr	**0**	**0**
TPMT*3C	c.719A>G	Tyr> Cys	**0**	**0**
**Total**	**-**	**-**	**112**	**100**

### Adverse effects

Collectively, 14 patients developed side effects associated with 6-MP therapy, characterized by rapidly developing severe myelosuppression expressed as leucopoenia alone or in combination with thrombocytopenia. Thirteen of those patients had wild type TPMT alleles and only one patient had a heterozygous TPMT*3A genotype.

The duration of 6-MP treatment doesn’t seem to significantly correlate with the occurrence of adverse effects by the time of sample collection (p-value = 0.87).

For the 14 patients with side effects, myelosuppression occurred at different time periods, ranging from 1–20 months after 6-MP administration (mean= 4.6 ± 5.2 months). On the other hand 6-MP treatment continued for 1–24 months (mean = 8.6 ±6.2) before samples were collected from the other patients and yet no evidence of myelosuppression was detected.

## Discussion

TPMT genotype influences the safety and efficacy of ALL treatment and genotype information may therefore be useful for optimizing 6-MP therapy [9,13,14]. Patients with both intermediate and absent TPMT activity have an increased risk of developing thiopurine-induced myelosuppression, compared with patients with normal activity [15]. For this reason, knowledge of the TPMT Single Nucleotide Polymorphism frequencies in a population is essential for estimating the proportions of risk groups under 6-MP therapy. From this point of view, in the current study, the frequencies of four variant TPMT alleles (TPMT*2, TPMT*3B, TPMT*3C, TPMT*3A), accounting for 80–95% of intermediate or low activity cases worldwide, were determined in pediatric ALL patients in Gaza Strip.

Only the TPMT*3A allele was detected in one patient out of 56 DNA samples from pediatric ALL patients. The frequency of TPMT*3A allele in Gaza strip is thus 0.89%. No TPMT*2, *3B or *3C alleles were detected. TPMT*3A allele frequency is consistent with ethnically related Israeli Arab subpopulations (0.79%) [16]. The frequency of TPMT*3A allele is also similar in Jordanian population (0.59) [17], Turkish population (0.9%) [18], and Iranian population (0.87%) [19]. It is lower than that reported for several Caucasian, African descendants and South American [17].

In the present study 14 patients developed myelotoxicity and they all had a normal TPMT genotype except for one patient who had the heterozygous TPMT*3A allele with rapidly developing severe myelosuppression. It is noteworthy to mention that not all cases of myelosupression are due to a mutation in the gene coding for the TPMT enzyme and therefore, not all cases can be prevented by screening for TPMT with either the enzymatic assay or genotype test [20]. The presence of toxicity in a number of cases and the lack of common types of mutations may result from the existence of other alleles, multigeneic contribution or other nongenetic factors [21]. Measurement of active 6-MP metabolite concentrations was suggested to be a key tool complementary to genotype in predicting toxicity under treatment with thiopurines [3].

In Gaza pediatric hospitals ALL patients are treated according to Berlin-Frankfurt-Mu¨nster protocol 2002 (BFM). Moreover protocols for treatment with 6-MP in local hospitals usually involve initial administration of low doses followed by gradual increase, but no TPMT or other metabolizing enzymes investigation are performed.

## Conclusions

TPMT*3A was the only deficiency alleles detected in the pediatric ALL patients in Gaza strip with an allelic frequency of 0.89%. Cases of myelosuppression in ALL pediatric patients treated with 6-MP in Gaza strip cannot be all explained by the existence of TPMT alleles (*2, *3A, *3B and *3C). Other polymorphic alleles in TPMT gene, or factors other than TPMT polymorphisms may be responsible for the development of toxicity.

## Competing interests

The authors declare that they have no competing interests.

## Authors’ contributions

BMA participated in the design of the study; designed and supervised on the molecular genetic studies and the statistical analysis and drafted the manuscript. WMH carried out and personally financed the molecular genetic studies and statistical analysis. AAA helped in supervision on the theoretical and practical work. All authors read and approved the final manuscript.

## Pre-publication history

The pre-publication history for this paper can be accessed here:

http://www.biomedcentral.com/2052-1839/13/3/prepub
